# The Best Colour for Sick-Room Walls

**Published:** 1886-11-20

**Authors:** 


### THE BEST COLOUR FOR SICK-ROOM WALLS.

WHAT is really the best colour or colours for the walls of a surgical ward? Is it green, or light blue, or ochre, or orange, or grey, or olive-green, or brick-red, or terra-cotta, or is it ordinary whitewash? Or is a variety best, or can any colour please, if a wall is unrelieved by pictures, or their equivalent? These questions it is not easy to answer shortly; but it is easy to name the physiological requirements of a surgical patient: in general he needs rest and grateful stimulation. For rest he must be free from all important irritating trifles—slight noises, ugly sights, inartistic bed-clothes, glaring pictures, harsh lights and shadows. He needs above all things soft light, and a variety of onlook. What is more depressing than dull light, and what irritates and maddens like a bare wall? Therefore our patient needs many windows, and a wall that he can rest his eyes on. Now white is tiresome as the snow; brick-red is like the meridian sun; grey is dispiriting as a mist; green is like spring-time, but fit only for nature in the spring; light blue, our own favourite, is too violent, requiring to set off the white clouds of July; and the only ones left are orange, ochre, terra-cotta, and olive-green. The last is mellow, soft, and grateful, fitting every time and place; and this, relieved by pine panelling, large windows, white coverlets, with no glaring oleograph pictures, seems to us the physiological best. Any pattern—as the case mentioned in THE HOSPITAL, No. 3—there should not be; unless it be so lightly laid on, and so indefinite that the individual pattern is lost in its general pleasing effects.

